# People with higher autistic traits show stronger binding for color–shape associations

**DOI:** 10.1038/s41598-023-36666-4

**Published:** 2023-06-13

**Authors:** Na Chen, Katsumi Watanabe, Charles Spence, Makoto Wada

**Affiliations:** 1 Department of Rehabilitation for Brain Functions, Research Institute of National Rehabilitation Center for Persons With Disabilities, Tokorozawa, 359-8555 Japan; 2 The Gonda Multidisciplinary Brain Research Center, Bar-Ilan University, Ramat Gan, 5290002 Israel; 3 Faculty of Science and Engineering, Waseda University, Tokyo, 169-8555 Japan; 4 Crossmodal Research Laboratory, University of Oxford, Oxford, OX2 6GG UK

**Keywords:** Psychology, Human behaviour

## Abstract

Non-synesthetes exhibit a tendency to associate specific shapes with particular colors (i.e., circle–red, triangle–yellow, and square–blue). Such color–shape associations (CSAs) could potentially affect the feature binding of colors and shapes, thus resulting in people reporting more binding errors in the case of incongruent, rather than congruent, colored-shape pairs. Individuals with autism spectrum disorder (ASD) exhibit atypical sensory processing and impaired multisensory integration. Here, we examined whether autistic traits ﻿(Autism-Spectrum Quotient; AQ) influence the strength of color–shape associations, as evidenced by the occurrence of binding errors in incongruent minus congruent conditions. Participants took part in an experiment designed to reveal binding errors induced by incongruent and congruent colored-shape pairs, and completed the Japanese version of the AQ score. The results revealed a significant correlation between AQ scores and occurrence of binding errors when participants were presented with the circle–red and triangle–yellow CSAs: That is, individuals with higher autistic traits tend to make more binding errors in incongruent minus congruent colored-shape pairs, indicating a stronger binding of circle–red and triangle–yellow associations. These results therefore suggest that autistic traits play a role in forming color–shape associations, shedding light on the nature of both color–shape associations and autistic perception.

## Introduction

### Color–shape associations (CSAs)

Color–shape associations (CSAs) refer to the naturally-biased associations that people have been documented to experience between specific colors and particular shapes. When asked to match a geometric shape with a color, people choose certain colors more frequently than others, leading to the existence of several widely-documented consensual color–shape associations (e.g., yellow–triangle)^1^. CSAs have captured the interest of artists and researchers for nearly a century, although it is only more recently that this phenomenon has been examined systematically^1–11^.

According to Chen et al.^1,12^, Japanese people exhibit systematic CSAs (﻿i.e., circle–red, triangle–yellow, and square–blue), which can be picked up using both direct (e.g., explicit color-shape matching tasks)^1^ and indirect (e.g., implicit association test; IAT)^12^ experimental methods. For instance, in the explicit color-shape matching task, participants were asked to choose the color that best matched a shape. The results showed that people chose red more often for circles, yellow more frequently for triangles, and blue more frequently for squares, with rates of 34.37%, 33.33%, and 19.40%, respectively, in contrast to the chance level of 12.5%^1^. Moreover, those naturally-biased CSAs could be explained by semantic sensory correspondences between colors and shapes (e.g., the shared semantics of “warm/cold” between colors and shapes)^1^. Thus, CSAs would appear to be acquired rather than innate. CSAs have been shown to influence visual processing when people discriminate and bind color and shape features^13^. That is, participants tend to respond more rapidly to color and shape features, when discriminating congruent color-shape combinations (i.e., red-circle, yellow-triangle, and blue-square) that share the same behavior responses (i.e., IATs)^12^, and when discriminating color and shape features following the priming of congruent CSAs (Go/No-go task)^13^. Participants make more binding errors following the visual presentation of incongruent (e.g., yellow-circle and red-triangle) than congruent (e.g., red-circle and yellow-triangle) colored-shape pairs^14^. Taken together, these findings suggest that CSAs constitute learned high-level associations, which could bias low-level visual perceptual processing, perhaps through a top-down effect.

Strictly-speaking, CSAs can be thought of as intramodal visual sensory correspondences, understood by reference to the emerging field of research on the crossmodal correspondences. Spence^15–17^ suggests four main hypotheses to explain synesthesia-like crossmodal correspondences: structural, statistical, emotion, and semantic correspondences. Structural correspondences occur when the neural system codes information from different sensory channels in adjacent brain areas or in similar ways (e.g., loudness–brightness)^18^. Statistical correspondences are acquired through perceptual learning with repeated exposure to natural co-occurrences (e.g., sound–elevation, pitch-size)^19^. Emotional correspondences arise from the emotional associations that people have with particular sensory stimuli (e.g., music–color)^20,21^. Semantic (or perhaps better-said linguistic) correspondences refer to the associations developed from ﻿the linguistic terms or semantic information underlying these sensory properties (e.g., possibly pitch–elevation, where the same linguistic terms are used e.g., ‘high’ / ‘low’)^22^. Among them, semantic/emotional sensory correspondences between colors and shapes have been suggested to account for CSAs (e.g., both visual features being associated with the same “warm/cold”, preference, and/or arousal values)^1–3,5,6^. However, CSAs may also be mediated by other structural/ statistical correspondence factors that have yet to be articulated.

### Feature integration theory

It has long been recognized that colors and shapes are registered and processed by discrete brain areas^23^, raising the question of how the unified binding of features associated with specific visual objects takes place^24^. According to the predominant theory of feature integration, ﻿visual features are initially processed in parallel in separate feature maps, and later bound together through spatial attention and/or top-down processes to their common locations^25–28^. Beyond perceiving, the memory system also maintains the specific conjunctions between individual features belonging to the same object/stimulus. Wheeler and Treisman^29^ proposed that binding is selectively stored if task-relevant, which is dependent on sustained attention, and when attention is withdrawn or distributed too widely, the joint object representations may break down into individual features. As such, multiple opportunities for errors, both perceptual in nature and memory-based (i.e., forgetting), arise when it comes to distinguishing an individual feature. Previous studies observed that responses are not only dispersed around the reported feature of the target, but are also clustered around the features of other non-target items from the perceptual array^30–32^. Reporting the feature value of a non-target item is often referred to as a “swap” error, reflecting a failure to retrieve the correct item from memory for the target feature^30^. Binding errors (i.e., swap errors)^33^ indicate a failure in binding and provide an important insight into the strength of feature binding.

Chen and Watanabe^34^ investigated the congruency effect of CSAs on binding errors. They found that CSAs influenced feature binding, with incongruent colored-shape pairs (e.g., yellow-circle and red-triangle) being subject to more binding errors than congruent colored-shape pairs (e.g., red-circle and yellow-triangle), indicating a stronger binding of congruent color–shape combinations. Regardless of whether performance errors in Chen and Watanabe’s study^34^ reflect perceptual or memory errors, they nevertheless both support the claim that the binding of congruent CSAs are stronger than incongruent ones. The congruency effect of CSAs on biasing feature binding may result from a top-down effect of CSAs, or CSAs occurring earlier before binding process and consequently biased binding. However, little is known concerning the factors related to the effect of CSAs on feature binding.

### Autism Spectrum Disorder (ASD)

Autism spectrum disorder (ASD) is a pervasive neurobiological developmental disorder, characterized by difficulties in social interaction and communication, restricted and repetitive behaviors/interests/activities, and atypical sensory behaviors^35^. Sensory symptoms, including hyper- or hypo-sensitivity to sensory stimuli, and deficits in multisensory integration, have been documented to occur in over 90% of autistic individuals^36,37^. A growing body of scientific evidence suggests that abnormalities in low-level visual perception and attention may account for the core deficits ﻿such as problems in social communication and repetitive behaviors^38,39^. Moreover, individuals with ASD have been shown to exhibit impaired multisensory integration and show less pronounced crossmodal correspondences. For example, the well-known “Bouba–Kiki” effect (i.e., people associate rounded shapes with words like “bouba” or “maluma,” and spiky shapes with words like “kiki” or “takete”)^40^ was weaker in individuals with ASDs than in controls^41–44^. Oberman and Ramachandran^43^ found that children with ASD do not exhibit the “bouba–kiki” effect that neurologically typical adults and children show (the neurotypical children exhibited 88% agreement on the “bouba–kiki” effect, as compared to only 56% for those with ASD, which is not significantly different from chance (50%)). Chen et al.^34^ reported that individuals with higher autistic traits showed fewer consensual color–taste (e.g., yellow–sour taste) and CSAs using a questionnaire survey. ﻿Hidaka and Yaguchi^45^ found that people with lower autistic traits exhibited a stronger cross-modal congruency effect for brightness-loudness associations in the speeded visual classification task, suggesting that they experienced stronger brightness-loudness associations than did those with higher autistic traits. Pellicano and Burr^46^ proposed a Bayesian model, suggesting that the attenuated Bayesian priors (hypo-priors), a tendency to perceive the world more accurately rather than modulated by prior experience, could perhaps explain the unusual sensory experience in ASDs. They suggested that the current sensory input is given proportionally greater weight than the learned priors, which may result in the difficulties in understanding the relationships of cross-sensory inputs and consequently lead to atypical multisensory integration^37,46,47^. Thus, it is possible that individuals with higher autistic traits may experience interference in their statistical learning, which could influence their learned CSAs. Specifically, they may show weaker binding in the case of congruent CSAs.

To our knowledge, there is no study that examined the effect of autistic traits on CSAs. Learning the influence of autistic traits on CSAs may help shed light on the nature of CSAs and improve our understanding of visual perception in individuals with ASD. It has been suggested that ASD represents the extreme end of the quantitative distribution of autistic traits in the general population^48^. The Autism-Spectrum Quotient (AQ) has been developed as a measure to assess autistic traits among typical-intelligence individuals both with and without ASD diagnoses^48,49^. Higher AQ scores indicate a greater magnitude of autistic traits, and individuals diagnosed with ASD tend to have larger AQ scores.

### The current study

Based on those previous findings, the aim of the current study was to explore the relationships between the strength of CSAs (tested by examining the nature of any binding errors that were induced by CSAs) and the degree of autistic traits using the AQ among Japanese participants. Using an experimental paradigm designed to reveal the strengths and binding errors associated with maintaining color–shape combinations, the participants were presented with brief displays of paired incongruent or congruent colored-shapes under conditions of divided attention, and reported the color of one of the two shapes^14^. The errors of mistakenly reporting the color of the adjacent shape presented in the display were defined as binding errors. Our previous study showed that participants made more binding errors in incongruent colored-shape pairs than congruent ones^14^. The differences of those binding errors between incongruent minus congruent colored-shape conditions were calculated as binding errors induced by each pair of CSAs. The binding errors may represent the strength and sensitivity of binding of CSAs. The autistic traits were measured by a Japanese version of the autism spectrum quotient (AQ-50) questionnaire survey^49^. If the binding errors induced by CSAs were found to correlate with AQ scores, it might suggest that autistic traits play a role in the binding of CSAs; if not, the null result may be taken to suggest little effect of autistic traits on CSAs. We hypothesized that autistic traits could affect CSAs that people with higher autistic traits exhibit a weaker binding in the case of CSAs, and we obtained results contrary to our hypothesis. Furthermore, the congruency effect of CSAs on binding errors was examined to replicate our previous study^14^.

## Method

### Participants

Fifty-one residents of Japan (twenty-four males, *M* age = 23 years, *SD* = 4.22) recruited from research participant pool of the National Rehabilitation Center for Persons with Disabilities took part in the experiment. A prior power analysis with G*power 3 determined that a sample of 44 individuals would be sufficient to detect a correlation coefficient of 0.4 with an alpha of 0.05, and a power of 80%^50,51^. We collected more data in the case of some participants' poor performance (e.g., making more than 50% errors in the letter task). None of the participants reported experiencing synesthesia and all had normal or corrected-to-normal visual acuity and normal color vision by self-report. Two participants who made more than 50% errors in the letter task were excluded, thus the data from forty-nine participants were used for data analysis. Ten participants were diagnosed with ASDs by a medical doctor (see Supplementary Table S1). The sample size of participants diagnosed with ASDs was chosen on the basis of a recent study investigating the effect of autistic traits on sensory processing^52^, and the availability of respondents from participants. Those participants with ASD received the Japanese version of the Autism Diagnostic Observation Schedule Component, Second Edition (ADOS-2)^53,54^ and a Japanese version of the Wechsler Adult Intelligence Scale-III^55,56^. This study was reviewed and approved by the ethics committee of the National Rehabilitation Center for Persons with Disabilities (2020–134; 2021–001) and conducted in accordance with the ethical standards laid down in the Declaration of Helsinki. Written informed consent was obtained from all participants in advance.

### Apparatus and stimuli

We adopted the experimental paradigm used in our previous study^14^. The experiment was programmed in E-Prime 2.0 (Psychology Software Tools; http://pstnet.com/products/e-prime/). The stimuli were displayed on a 24-inch LED monitor (EIZO CS2410, EIZO Corp, Hakusan, Japan), with a 1920 × 1080-pixel resolution and a refresh rate of 100 Hz. Participants viewed the monitor at a distance of approximately 60 cm.

**Figure 1:**
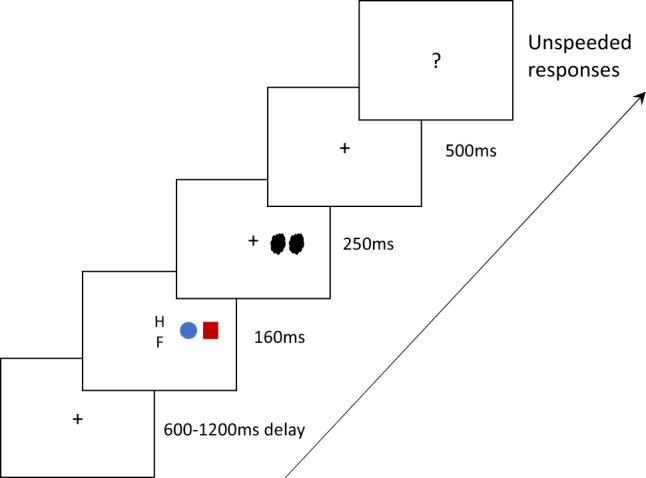
An example of the trial sequence with an incongruent trial. Following a variable interval, two letters appeared in the middle of the screen together with two colored-shapes presented to either the left or the right side of the letters. At the end of the trial, participants were required to give three responses by key press (same or different letter, the color of one particular shape (e.g., “What was the color of the circle?”), and a confidence rating concerning their color choice).

Nine colored-shapes and two black letters were used as stimuli. Three colors (i.e., red, yellow, blue) and three shapes (i.e., circle, triangle, square) generated nine colored-shape combinations. The three colors were measured by PR-655 (Photo Research, Chatsworth, CA, USA) and each color was measured 10 times and the average calculated. The color information was as follows: Yellow: L* = 80.16, a* = -4.07, b* = 60.20; Red: L* = 74.07, a* = 100.7, b* = 36.70; Blue: L* = 79.48, a* = -24.59, b* = -94.51. The three shape stimuli were all black line drawings. The circle was 2.1° in diameter, the square was 1.8° (in height) × 1.8° (in width), and the triangle was 2.4° (in height) × 2.1° (in width). The three shape stimuli were presented in the upward orientation against a white background. The two colored-shapes were horizontally aligned, and the shape near the center of the screen maintained a distance of 10° of visual angle to the left or to the right side of the screen. The two colored-shapes were separated by 2.6° of visual angle. Two black letters served as fixation stimuli (H and F) appeared at the center of the screen and subtended ~ 0.7° × 0.7° of visual angle. Two hash signals served as the masks for the colored-shapes (see an example in Fig. 1). They were printed in black, and were presented at ~ 1.8° × 1.8° of visual angle.

### Procedure

The experiment was carried out in a laboratory with the lighting dimmed. The structure of the study was based on the previous study^14^. Following a variable interval (from 600 to 1200 ms), the stimulus display was presented for 160 ms, consisting of the simultaneous presentation of two letters at the center of the screen and two colored-shapes presented either to the left or to the right side of the letters (see Fig. 1). The letters were presented vertically and were either the same (i.e., H/H, F/F) or different (i.e., H/F, F/H). Following the offset of the stimulus display, two pattern masks consisting of two hash symbols were presented for 250 ms where the two colored-shapes had been presented. The presentation of the masks was followed by a blank with a fixation of 500 ms. The participants had to make three responses. First, they reported whether the two letters presented at the center of the display were the “same” or “different”. Second, they reported the color of one of the two shapes that had been presented (e.g., what was the color of the circle in Fig. 1). Finally, they were required to indicate their confidence level regarding their color choice, using a scale from 1 to 6 (1 = least confident, 6 = most confident). The order of the responses was kept constant during the session. Response options were given by labeled keys on the computer keyboard. The letter *u* and *i* were labeled as “same” and “different”, respectively; the letters *j*, *k*, and *l* were labeled red, yellow, and blue, respectively. The confidence response was given by pressing the numbers 1–6 on the keyboard.

The two shapes presented on each trial were always different and randomly chosen from the three shapes (i.e., circle, triangle, square), giving rise to six possible combinations of two shapes. There was one within-subject factor, the color–shape congruency concerning the Japanese CSAs (i.e., circle–red, triangle–yellow, and square–blue). Therefore, for each pair of shapes, the color-shape assignments were either congruent or incongruent with the color–shape associations, leading to 12 possible colored-shape combinations, combined with the two shapes, giving rise to a total of 24 combinations. Taking the circle and triangle pair as an example, the colored-shape pairs were either congruent (circle–red/triangle–yellow pairs) or incongruent (circle–yellow/triangle–red pairs; the opposite of congruent pairs). The other colored-circle or colored-triangle stimulus (e.g., a blue–circle or blue–triangle) was not presented. Then, for each colored-shape pair that had been presented, the participants were asked about the color of each of the shapes in a random order (e.g., “What was the color of circle?” and “What was the color of square?” in the example shown in Fig. 1). Finally, the position of the colored-shape stimuli could be on the left or right to the screen center. With the first question on letter conditions, we generated three conditions (i.e., (H/H, F/F), H/F, F/H). In all, there could be 24 (colored-shape combination) × 2 (color choice for one shape) × 2 (stimuli location) × 3 (letter condition) = 288 trials. The experiment trials were preceded by 30 practice trials. The 288 trials were broken into six blocks of 48 trails. At the end of each block, participants took a self-determined break. The whole experiment took approximately 45 ~ 60 min.

After the behavioral experiment, participants were instructed to complete a paper questionnaire survey examining their explicit CSAs^57^. Outlines of the three shapes (i.e., circle, triangle, and square) were presented in vertical on the left side of the page. A color wheel filled with four colors (i.e., red, yellow, blue, and green) was presented next to each of the shapes (See Supplementary Figure S1). The order of the shapes and the rotation of the color wheel were counterbalanced across participants. The participants were asked to choose the color that best matched each shape from the four-color wheel. Note that participants could choose the same color for several shapes (e.g., red-circle and red-triangle).

### Autism Spectrum Quotient

**Figure 2:**
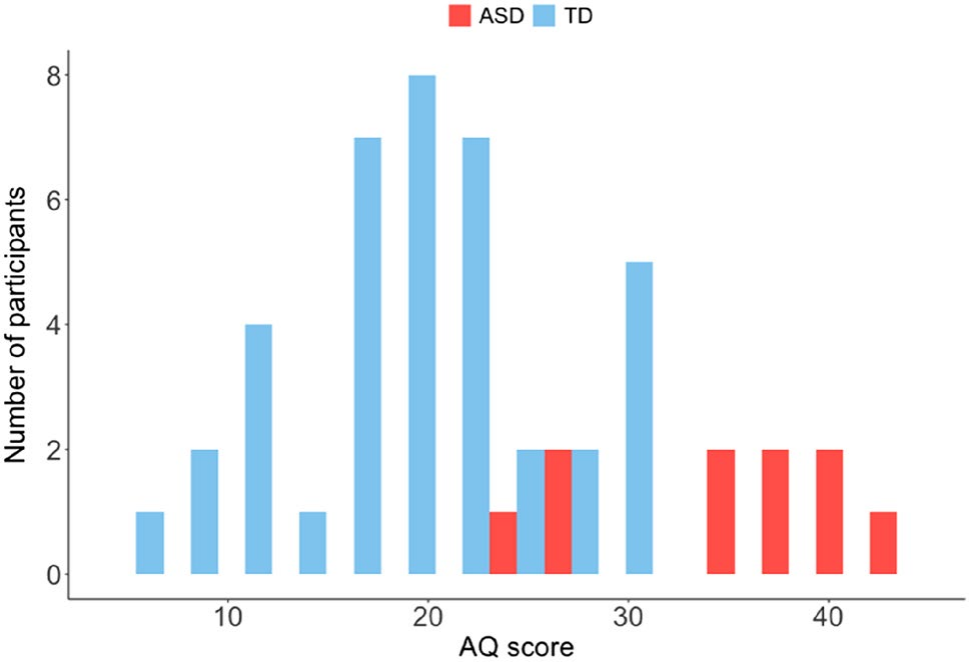
Distribution of participants’ AQ scores.

Finally, the participants completed a Japanese version of the AQ developed by Wakabayashi et al.^58^ based on an original version^48^. The AQ is a widely used self-reported questionnaire to measure autistic traits in the general population^48,49^. Distribution of the total AQ scores for the 49 participants is shown in Fig. 2.

### Analysis

There were three possible outcomes for reporting the color of the shape: Hit, binding error (BE), and feature error (FE)^14^. Taking the stimuli displayed in Fig. 1 as an example, when the question asks for the color of the circle, a hit is correctly reporting the target color (i.e., “blue”), an BE is reporting the color of the distractor (i.e., “red”), and an FE is reporting a color not presented in the display (i.e., “yellow”). We calculated the mean proportion of hits, BEs, and FEs (number of trials × 100%/ 288 trials) for each participant with color–shape congruency factor (congruent CSAs: circle–red, triangle–yellow, and square–blue). A paired-sample *t*-test with the Bonferroni correction (the significance level was 0.05/3 = 0.017) was used to examine the congruency effect of CSAs on hits, BEs, and FEs for the three colored-shape pairs, separately. To further quality the results, the Bayes Factors (BF10) were referred to, in order to determine whether or not there was support in favor of the alternative (H1) or null (H0) hypotheses^59^.

BEs induced by each pair of CSAs were calculated by the difference of BEs in incongruent minus congruent colored-shape pairs (i.e., BEs induced by each of the three pairs of CSAs = (BEs in incongruent-CSA – BEs in congruent-CSA).

The AQ scores were normally distributed in the current sample (*W* = 0.97, *p* = 0.25; Shapiro–Wilk’s test). Pearson’s correlation analysis was used to examine the relationships between the total AQ scores and the BEs induced by each pair of CSAs.

To further understand the effect of autistic traits on BEs induced by CSAs, participants with typical development (TD) were divided into high-AQ and low-AQ groups. In order to balance the sample size, the medium value of AQ score (AQ = 20) in the current sample was used as the criterion for the high (AQ ≥ 20, *n* = 20) and low (AQ < 20, *n* = 19) AQ groups^60^. A one-way ﻿Analysis of Variance (ANOVA) was used to compare the BEs induced by CSAs in the three groups. Data analysis were performed using R 4.0.2 software^61^.

## Results

### Mean proportion of BEs, hits, and FEs

**Figure 3:**
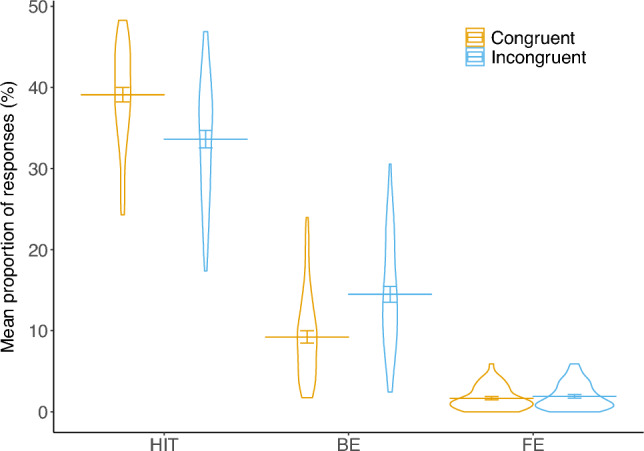
Violin plot describing the distribution of individual responses of hits, BEs, and FEs in congruent and incongruent conditions. Each dot represents the mean response in each condition for an individual participant. The horizontal line represents the mean. Error bars represent the standard errors of the mean. *BE *binding error, *HIT* correct responses, *FE* feature error.

Accuracy on the letter task was 93.96% (*SD* = 5.24%) and accuracy on the color task was 72.73% (*SD* = 12.91%). Figure 3 shows the congruency effect of CSAs on hits, BEs, and FEs. A paired-sample *t*-test revealed a significant difference in BEs between the congruent and incongruent conditions, *t*(48) = 7.42, Bonferroni corrected *p* < 0.01, Cohen’s *d* = 1.06, BF_10_ = 6,917,306, with participants making more BEs in the incongruent conditions (14.48%) than in the congruent conditions (9.23%; see Fig. 3). For hits, there was also a significant difference, *t*(48) = 7.96, Bonferroni corrected *p* < 0.01, Cohen’s *d* = 1.14, BF_10_ = 41,153,391, with participants making significantly more hits on the congruent (39.11%) than on the incongruent trials (33.62%). FEs did not differ between the congruent (1.67%) and incongruent conditions (1.90%), *t*(48) = 1.86, Bonferroni corrected *p* = 0.21, Cohen’s* d* = 0.27, BF_10_ = 0.76.

### BEs for each of the color–shape pairs

**Figure 4:**
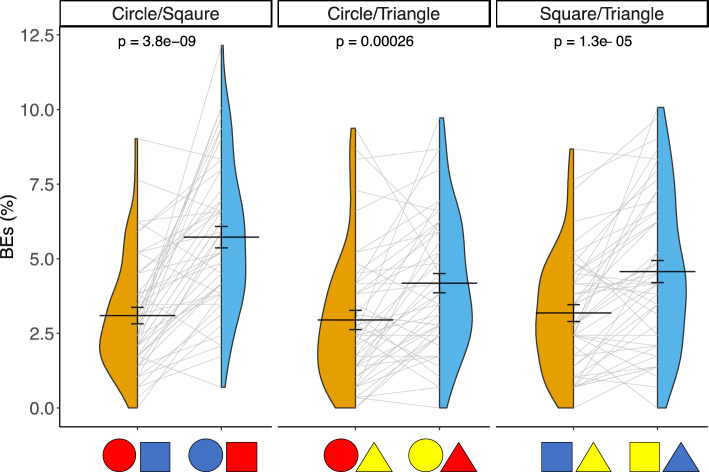
Violin plot showing the distribution of individual BEs in the congruent and incongruent conditions for each of the three colored-shape pairs. The horizontal line represents the mean. Error bars represent the standard errors of the mean. *p* values are calculated from paired-sample *t*-tests (all *p*s < .001).

Next, we further explored the BEs in the congruent and incongruent conditions by examining each pair of colored-shape combinations separately (e.g., for the circle/triangle pair, congruent: circle–red/triangle–yellow; incongruent: circle–yellow/triangle–red; Fig. 4). Paired sample *t*-tests revealed that for each colored-shape pair, the BEs in the incongruent condition were significantly larger than were those in the congruent condition (circle/square pair, 5.73% vs. 3.10%, *t*(48) = 7.18, Bonferroni corrected *p* < 0.01, *d* = 1.03, BF_10_ = 3,131,724; circle/triangle pair, 4.18% vs. 2.95%, *t*(48) = 3.95, Bonferroni corrected *p* < 0.01, *d* = 0.56, BF_10_ = 97.82; triangle/square pair, 4.57% vs. 3.18%, *t*(48) = 4.86, Bonferroni corrected *p* < 0.01, *d* = 0.69, BF_10_ = 1521.45). Therefore, BEs occurred more frequently in the incongruent conditions than in the congruent conditions for all three of the colored-shape pairs, consistent with Chen and Watanabe’s previous findings^14^.

### Correlation of BEs induced by CSAs and AQ scores

**Figure 5:**
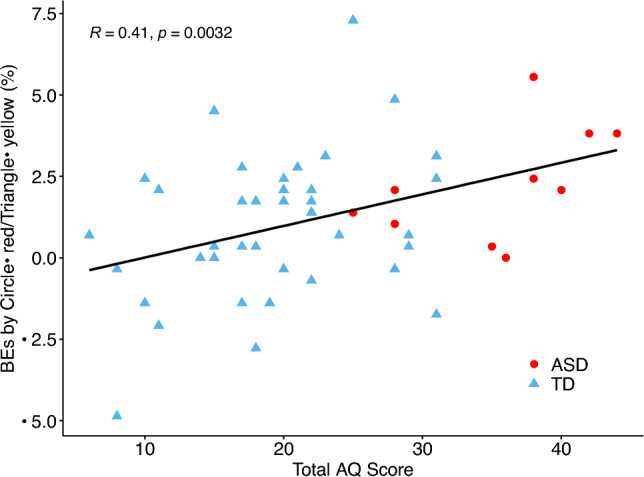
Correlation between total AQ scores and BEs induced by circle–red and triangle–yellow associations.

Pearson’s correlation analysis showed a significant correlation between the total AQ scores and BEs induced by circle–red and triangle–yellow associations (*r* = 0.41, *p* = 0.003, BF_10_ = 15.80; Fig. 5). No significant correlations were observed between total AQ scores and BEs induced by circle–red and square–blue associations (*r* = -0.12, *p* = 0.41, BF_10_ = 0.44), or between total AQ scores and BEs induced by triangle–yellow and square–blue associations (*r* = -0.04, *p* = 0.79, BF_10_ = 0.33).

**Figure 6:**
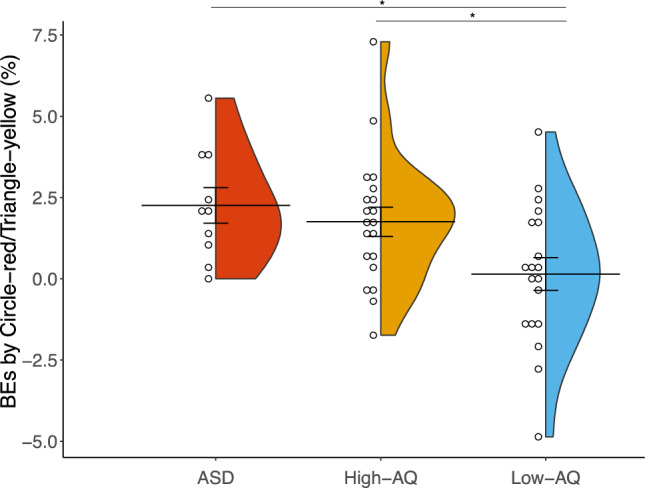
Violin plot showing the distribution of BEs induced by circle–red and triangle–yellow associations in the ASD, High-AQ, and Low-AQ groups. Each dot represents the mean BE induced by circle–red and triangle–yellow for an individual participant. The horizontal line represents the mean. Error bars represent the standard errors of the mean. (^*^p < 0.05).

A one-way ANOVA showed a significant difference on BEs induced by circle–red and triangle–yellow associations between the three groups of participants, *F*(2, 46) = 4.62, *p* = 0.015, η_p_^2^ = 0.17 (Fig. 6). ∆BEs in ASD (mean = 2.26, *sd* = 1.73; *p* = 0.029) and high-AQ (mean = 1.75, *sd* = 2.02; *p* = 0.045) groups were significantly larger than those in the low-AQ group (mean = 0.15, *sd* = 2.19), and there was no difference between the ASD and high-AQ groups (*p* = 0.80). Moreover, for the ASD and high-AQ groups, the ∆BEs were significantly greater than zero (ASD: *t*(9) = 4.13, Bonferroni corrected *p* = 0.009, Cohen’s *d* = 1.31, BF10 = 18.85; High-AQ group: *t*(19) = 3.88, Bonferroni corrected *p* = 0.003, Cohen’s *d* = 0.87, BF10 = 36.06), while there was no difference between the ∆BEs and zero in the low-AQ groups (*t*(18) = 0.29, Bonferroni corrected *p* > 1, Cohen’s *d* = 0.07, BF10 = 0.25). Thus, participants with ASD and high-AQ scores tended to show more BEs induced by circle–red and triangle–yellow associations, indicating a stronger binding of circle–red and triangle–yellow associations. Those results were consistent with the findings from correlation analysis.

Moreover, there was no significant correlation between the total AQ scores and the sum of BEs (BEs in congruent plus incongruent colored-shape pairs) for each of three colored-shape pairs (circle/square: *r* = -0.18, *p* = 0.22, BF_10_ = 0.64; circle/triangle: *r* = -0.04, *p* = 0.77, BF_10_ = 0.33; square/triangle: *r* = -0.21, *p* = 0.14, BF_10_ = 0.85). Furthermore, no significant correlation was observed between total AQ scores and the sum of BEs, hits, and FEs across the three colored-shape pairs (BEs:* r* = -0.15, *p* = 0.29, BF_10_ = 0.53; hits: *r* = 0.16, *p* = 0.26, BF_10_ = 0.57; FEs: *r* = -0.14, *p* = 0.33, BF_10_ = 0.49).

### Hits and BEs as a function of confidence ratings

Next, we examined the confidence ratings for the reported color choices. First, we classified confidence ratings as low (answering 1 or 2), medium (answering 3 or 4), and high (answering 5 or 6). As there were far fewer FEs (3.56%), we excluded FEs from this analysis.

A two-way confidence level (3) × congruency conditions (2) ANOVA on the mean proportion of hits was conducted. The results revealed a significant main effect of congruency condition, *F*(1, 254) = 4.02, *p* = 0.046, η_p_^2^ = 0.01, a significant effect of confidence level, *F*(2, 254) = 19.84, *p* < 0.01, η_p_^2^ = 0.13, but no significant interaction, *F*(2, 254) = 0.12, *p* = 0.89, η_p_^2^ = 0.001. Participants made more hit responses in the congruent (15.0%) than in the incongruent (12.5%) conditions. The participants made more hits in high and medium confidence trials than in low confidence trials (﻿with 8.6%, 15.6%, and 17.6% for low, medium and high confidence levels, respectively; all *p*s < 0.01), and there was no difference between high and medium confidence level (*p* = 0.40).

A two-way ANOVA for BEs revealed a significant main effect of congruency condition, *F*(1, 238) = 10.24, *p* = 0.002, η_p_^2^ = 0.04, a significant main effect of confidence level, *F*(2, 238) = 5.07, *p* = 0.007, η_p_^2^ = 0.04, and no significant interaction, *F*(2, 238) = 0.42, *p* = 0.66, η_p_^2^ = 0.003. Participants made more BEs in the incongruent (5.72%) than in the congruent (3.77%) conditions. Participants made fewer BEs in high (3.27%) than low (5.42%) and medium (5.35%; *p*s < 0.05) confidence levels. There was no difference between low and medium confidence ratings (*p* = 1).

### Explicit questionnaire on CSAs

The frequency of color choice for each shape were shown in Table 1. Chi-square test showed significant associations of colors for each shape [circle: *x*(3)^2^ = 64.22, *p* < 0.01; triangle:* x*(3)^2^ = 36.31, *p* < 0.01; square:* x*(3)^2^ = 25.69, *p* < 0.01]. ﻿The adjusted residual analysis showed that red was chosen for circle more frequently than the other colors (*z* = 7.84, *p* < 0.01), yellow for triangle (*z* = 5.86, *p* < 0.01), and blue for square (*z* = 4.87, *p* < 0.01). Those results were consistent with previous findings^1,12^.

**Table 1:** The choosing frequency of colors for shapes (%).

	Circle	Triangle	Square
Red	***73.47***	20.41	8.16
Yellow	18.37	***61.22***	14.29
Blue	6.12	12.24	***55.10***
Green	2.04	6.12	22.45

Values in bold indicate a significant association between color and shape, *p* < 0.01.

Further analysis divided participants into two groups, based on those participants who chose the matched associations for circle–red and triangle–yellow (*N* = 27) and those who chose the not-matched combinations of associations for circle and triangle (*N* = 22). Linear mixed model analysis using the association group (2; Matched and not-matched) and autistic traits group (3; “ASD”, “High-AQ”, and “Low-AQ”), and the interaction effect as fixed factors, and participants as random factor on the ∆BEs in circle and triangle pairs, showed that there was no interaction effect between the matching-association group and autistic traits group [*x*(2)^2^ = 0.70, *p* = 0.70], indicating that autistic traits group might not influence the explicit associations for circle–red and triangle–yellow. Significant main effects of autistic traits group [*x*(2)^2^ = 9.19, *p* = 0.01] and association group [*x*(1)^2^ = 5.49, *p* = 0.02] were observed. BEs by circle–red and triangle–yellow associations were higher in people (mean = 1.86, *sd* = 0.44) who explicitly chose the circle–red and triangle–yellow associations than people (mean = 0.46, *sd* = 0.38) who chose the other associations for circle and triangle.

## Discussion

In the present study, we examined whether autistic traits influenced Japanese CSAs^1^ tested by binding errors. The results revealed a significant correlation between total AQ scores and BEs induced by circle–red and triangle–yellow associations. Participants with ASD and those with high AQ scores made more BEs in the incongruent than in the congruent conditions for circle and triangle shape pairs, compared with the participants with a low AQ score. Those results indicate that participants with higher autistic traits are more likely to bind circle–red and triangle–yellow combinations, which is opposite to the hypothesis that people with higher autistic traits exhibit a weaker binding in the case of CSAs. Moreover, participants made more BEs in the incongruent than in the congruent conditions for all the three colored-shape combinations, replicating our previous findings^14^. Those results demonstrated that autistic traits play a role in the binding of congruent CSAs, that people with higher autistic traits exhibit stronger binding of specific CSAs.

### Binding errors for CSAs

When two colored-shape stimuli were presented shortly and closely in the periphery visual field under conditions of divided attention, participants made more BEs reporting the color of one of the two shapes in the incongruent conditions than congruent conditions in line with CSAs^1^. Thus, CSAs biased the feature binding of colors and shapes, with congruent colored-shape combinations showing stronger binding than incongruent ones when recalling from memory. The higher occurrence of BEs for the incongruent than congruent colored-shape pairs suggest a top-down effect of CSAs, or possibly that some CSAs occur prior to binding. Previous studies have suggested that CSAs are learned semantic sensory correspondences, that shared semantic information describing colors and shapes could explain those CSAs (e.g., warmth/lightness)^1,2^. Those learned high-level CSAs could play a top-down effect to bias the low-level perceptual and memory processing, and modulate the binding of color and shape features^62–64^. Those results are consistent with previous findings that stored knowledge of color–shape conjunctions could modulate binding, which may occur at an early stage of processing, and require modest attention (e.g., yellow for a banana-like shape)^65–70^. The occurrence of BEs may reflect both perceptual mis-combination and memory-based errors. To correctly recalling of color for a shape require the memory strength/sensitivity for the colored-shape combinations. Participants made more hits (i.e., correctly report of color) and less BEs in congruent than incongruent colored-shape pairs, suggesting that congruent colored-shape combinations are easier to be memorized and recalled.

### Effect of autistic traits on CSAs

Here, we further demonstrated that the BEs induced by certain CSAs (i.e., circle–red and triangle–yellow) were significantly correlated with AQ scores, that participants with higher autistic traits made more BEs in incongruent than congruent conditions for circle and triangle stimuli pairs, indicating a stronger binding of circle–red and triangle–yellow associations in those participants. According to previous research, CSAs could be innate, or emerge at an early age^71–76^. For instance, in a forced choice task, 2.5-year-old toddlers are preferentially disposed to pair certain shapes with specific colors^75^. A related disposition has been evidenced in even younger infants (aged 2–3 months)^76^. It has been proposed that early development is a period of exuberant neural connectivity that facilitate arbitrary sensory experience, similar to the sensory experience of adults with synesthesia (i.e., simulation of one sense involuntarily evokes another sense)^77^, and dissipates during development^76,78^. CSAs might also emerge earlier in infants. Spector and Maurer^75^ reported that pre-literate children (30–36 months) associated letters of the alphabet with colors based on their shape (e.g., O-white, X-black), and did not show other associations (e.g., B-blue, A-red) seen in literature children (7–9 years) and adults which might be based in language (e.g., B is the first letter of blue) and semantic associations. They suggested that certain color–shape associations are innate, that sensory cortical organization initially binds color to some shapes. Our previous studies showed that children at the age of 6 ~ 8 years of age could show similar patterns of CSAs as adults^71,72^. Those results may indicate that once a prior association is formed in individuals with higher autistic traits, it is likely to remain strongly associated, and tended to be less easily updated by later development, resulting in a hypo-prior^46,47,52,75^. Pellicano and Burr^46^ suggested a Bayesian model for autistic perception, that people with autism weight their prior experience less than do neurotypical individuals, leading to a tendency to perceive the world more accurately because less influenced by learned top-down influences. It might be possible that CSAs are innate or emerged earlier in life^70–75^, and individuals with higher autistic traits updated little by statistical learning with color and shape conjunctions from the environment in the later development, resulting in less top-down influence and more bottom-up effect on binding of congruent CSAs. However, CSAs in participants with lower autistic traits could be interfered and updated with statistical associations of color and shape features, which exert more top-down effect and weaken the original binding of congruent CSAs.

Meanwhile, we observed little effect of autistic traits on hits, BEs, and FEs, and little effect of autistic traits on BEs in the three colored-shape combinations, only the effect of autistic traits on biasing the BEs correlated by CSAs (i.e., circle–red, triangle–yellow) was significant. Previous studies suggested that individuals with ASD exhibited atypical sensory processing and difficulties in binding sensory information into a unified percept^78,79^. Some studies also reported enhanced visual perceptual abilities in individuals with ASD (e.g., color discrimination, visual search tasks)^80,81^. For instance, Stevenson et al.^33^ reported both enhanced sensory processing in terms of accuracy and precision of sensory recall, and increased binding errors of color and spatial location in children with ASD. However, this study showed little effect of autistic traits on the visual working memory of color recall. Thus, the stronger binding of CSAs in participants with higher autistic traits may be little influenced by the effect of autistic traits on low-level visual processing.

The stronger binding of circle–red and triangle–yellow associations in individuals with higher autistic traits may also suggest that CSAs have some structural correspondence basis shaped by the neurological coding system^67^. Studies suggested that individuals with ASD have different connectivity between brain regions in general, and showed atypical brain activity or connectivity when performing visual perceptual tasks, such as relative hyperactivation of the early visual cortex in individuals with ASD in visuospatial tasks, attention shifting tasks^82,83^. The excessively activated low-level visual processing in individuals with ASD might underline a stronger binding of some CSAs in participants with higher autistic traits. Future studies are needed to further explore the brain patterns of connectivity and activity on congruency effect of CSAs.

Moreover, triangle–yellow and circle–red associations are stronger than the square–blue mapping in Japanese participants (triangle–yellow: 33%; circle–red: 34%; square–blue: 19%; with a chance level of 12.5%)^1^. The strong circle–red association (especially amongst the Japanese participants tested here) may stem from the image of the Japanese flag. With a red circle, symbolizing the sun, placed on a rectangular white background, the national flag of Japan may play a critical role on the forming of circle–red association in Japanese people. Specifically, the white background used in the current study may have enhanced the resemblance to the red-circle in the flag, leading to a strong binding of red and circle^84^. In a previous study, we also observed that people with higher autistic traits showed fewer consensual associations between colors and familiar geometric shapes, which might be explained by a reduced prior knowledge effect in individuals with higher autistic traits^34^. When testing the strength of CSAs using behavioral experimental methods, those individuals with higher autistic traits showed stronger binding of CSAs. It may imply that CSAs have different mechanisms of correspondences, with some being innate, and structurally constructed, while some may be shaped by statistical/semantic learning experiences.

### Explicit CSAs

The explicit questionnaire survey showed further evidence for the CSAs observed in previous findings^12,57^. Moreover, participants who chose the matched CSAs showed stronger binding of circle–red and triangle–yellow than participants who chose the not-matched CSAs. There was no significant effect of participants’ group of autistic traits on the explicit CSAs, which may suggest that people are aware of CSAs when asked explicitly, but the effect of autistic traits on the strength of those CSAs could be observed by indirect experimental method.

It should be noted that the questionnaire on the explicit CSAs was performed after the main binding task. The color choices for shapes might be influenced by the priming effect of CSAs from the binding task, that the congruent color–shape combinations were more frequently presented than incongruent ones. Nevertheless, the current finding on explicit CSAs was consistent with the previous study using an explicit matching task^1^.

### Confidence ratings

The confidence ratings for reporting color choices revealed that people made more hits in high and medium confidence levels than low confidence levels, and participants made less BEs in high confidence levels than low and medium confidence levels, as might have been expected. These results suggest that our participants could be aware of their memory errors, that binding errors could be related to subjectively feeling of confidence levels^62,85^.

### Limitation

One limitation of the current study is that the congruent color–shape combinations were more frequently presented than incongruent ones, which may lead to a bias in response errors. According to the hypo-priors hypothesis^46^, participants with lower autistic traits may perform better at statistical learning with the stimuli set, and make fewer binding errors in congruent than incongruent conditions. However, opposite results were observed, that participants with higher autistic traits showed fewer binding errors at congruent than incongruent conditions, providing further evidence for the stronger binding for some CSAs.

### Summary

In summary, the results of the present study demonstrate that autistic traits correlate significantly with binding errors induced by circle–red and triangle–yellow associations, and that participants with higher autistic traits tended to show stronger binding of circle–red and triangle–yellow associations. Thus, autistic traits could affect the maintenance of CSAs, and hypo-priors hypothesis in autistic perception may explain these findings. Specific CSAs might be innate or constructed earlier in life, with stronger binding being preserved in those individuals with higher autistic traits, and being little influenced by later statistical learning. Future studies are in need to further examine the nature of CSAs by examining CSAs in infants and in blind individuals.

## Supplementary Information

Supplementary Information.

## Data Availability

The raw data and R code for the current study are available online, https://osf.io/mpjeu/.

## References

[1] Chen N, Tanaka K, Matsuyoshi D, Watanabe K (2015). Associations between color and shape in Japanese observers. Psychol. Aesthet. Creat. Arts.

[2] Albertazzi L, Da Pos O, Canal L, Micciolo R, Malfatti M, Vescovi M (2013). The hue of shapes. J. Exp. Psychol. Hum. Percept. Perform..

[3] Dreksler N, Spence C (2019). A critical analysis of colour–shape correspondences: Examining the replicability of colour-shape associations. i-Perception.

[4] Dumitrescu A (2011). New researches regarding relationship between elementary geometric shapes and basic colors. Ann. DAAAM Proc..

[5] Hanada M (2019). Associations of visual forms with colors: The minor role of emotion as the mediator. Color Res. Appl..

[6] Jacobsen T (2002). Kandinsky's questionnaire revisited: Fundamental correspondence of basic colors and forms?. Percept. Mot. Skills..

[7] Jacobsen T, Wolsdorff C (2007). Does history affect aesthetic preference? Kandinsky's teaching of colour-form correspondence, empirical aesthetics, and the Bauhaus. Des. J..

[8] Kandinsky W. On the spiritual in art. New York, NY: Da Capo Press.1912/1994.

[9] Kandinsky W. Point and line to plane. New York, NY: Solomon R. Guggenheim Foundation, First German Ed. 1947.

[10] Kharkhurin AV (2012). Is triangle really yellow? An empirical investigation of Kandinsky’s correspondence theory. Emp. Stud. Arts.

[11] Walter S, MacDonald LW, Biggam CP, Paramei GV (2018). Kandinsky’s colour-form correspondence theory. Progress in colour studies: Cognition, language and beyond.

[12] Chen N, Tanaka K, Watanabe K (2015). Color–shape associations revealed with implicit association tests. PLoS ONE.

[13] Chen N, Watanabe K (2023). Effect of Color-shape associations on visual feature discrimination. Q. J. Exp. Psychol..

[14] Chen N, Watanabe K (2021). Color–shape associations affect feature binding. Psychon. Bull. Rev..

[15] Spence C (2011). Crossmodal correspondences: A tutorial review. Attent. Percept. Psychophys..

[16] Spence C, Howes D (2018). Crossmodal correspondences: A tutorial review. Senses and sensation: Critical and primary sources.

[17] Spence C (2022). Exploring group differences in the crossmodal correspondences. Multisens. Res..

[18] Ramachandran VS, Hubbard EM (2001). Psychophysical investigations into the neural basis of synaesthesia. Proc. R. Soc. Lond. B. Biol. Sci..

[19] Parise CV, Knorre K, Ernst MO (2014). Natural auditory scene statistics shapes human spatial hearing. Proc. Natl. Acad. Sci..

[20] Palmer SE, Schloss KB, Xu Z, Prado-León LR (2013). Music–color associations are mediated by emotion. Proc. Natl. Acad. Sci..

[21] Spence C (2020). Assessing the role of emotional mediation in explaining crossmodal correspondences involving musical stimuli. Multisens. Res..

[22] Walker L, Walker P (2016). Cross-sensory mapping of feature values in the size–brightness correspondence can be more relative than absolute. J. Exp. Psychol. Hum. Percept. Perform..

[23] Rentzeperis I, Nikolaev AR, Kiper DC, van Leeuwen C (2014). Distributed processing of color and form in the visual cortex. Front. Psychol..

[24] Singer W, Gray CM (1995). Visual feature integration and the temporal correlation hypothesis. Annu. Rev. Neurosci..

[25] Quinlan PT (2003). Visual feature integration theory: Past, present, and future. Psychol. Bull..

[26] Treisman AM, Gelade G (1980). A feature-integration theory of attention. Cogn. Psychol..

[27] Treisman A (1996). The binding problem. Curr. Opin. Neurobiol..

[28] Treisman A (1998). Feature binding, attention and object perception. Philos. Trans. R. Soc. B..

[29] Wheeler ME, Treisman AM (2002). Binding in short-term visual memory. J. Exp. Psychol. Gen..

[30] Bays PM, Catalao RF, Husain M (2009). The precision of visual working memory is set by allocation of a shared resource. J. Vis..

[31] Oberauer K, Lin HY (2017). An interference model of visual working memory. Psychol. Rev..

[32] Schneegans S, Bays PM (2017). Neural architecture for feature binding in visual working memory. J. Neurosci..

[33] Stevenson RA, Ruppel J, Sun SZ, Segers M, Zapparoli BL, Bebko JM, Ferber S (2021). Visual working memory and sensory processing in autistic children. Sci. Rep..

[34] Chen N, Watanabe K, Wada M (2021). People with high autistic traits show fewer consensual crossmodal correspondences between visual features and tastes. Front. Psychol..

[35] American Psychiatric Association (2013). Diagnostic and Statistical Manual of Mental Disorders (DSM-5).

[36] DuBois D, Lymer E, Gibson BE, Desarkar P, Nalder E (2017). Assessing sensory processing dysfunction in adults and adolescents with autism spectrum disorder: A scoping review. Brain Sci..

[37] Robertson CE, Baron-Cohen S (2017). Sensory perception in autism. Nat. Rev. Neurosci..

[38] Dellapiazza F, Vernhet C, Blanc N, Miot S, Schmidt R, Baghdadli A (2018). Links between sensory processing, adaptive behaviours, and attention in children with autism spectrum disorder: A systematic review. Psychiatry Res..

[39] Thye MD, Bednarz HM, Herringshaw AJ, Sartin EB, Kana RK (2018). The impact of atypical sensory processing on social impairments in autism spectrum disorder. Dev. Cogn. Neurosci..

[40] Köhler W (1947). Gestalt psychology.

[41] Gold R, Segal O (2017). The bouba-kiki effect and its relation to the autism quotient (AQ) in autistic adolescents. Res. Dev. Disabil..

[42] Król ME, Ferenc K (2020). Silent shapes and shapeless sounds: The robustness of the diminished crossmodal correspondences effect in autism spectrum conditions. Psychol. Res..

[43] Oberman LM, Ramachandran VS (2008). Preliminary evidence for deficits in multisensory integration in autism spectrum disorders: The mirror neuron hypothesis. Soc. Neurosci..

[44] Occelli V, Esposito G, Venuti P, Arduino GM, Zampini M (2013). The Takete—Maluma phenomenon in autism spectrum disorders. Perception.

[45] Hidaka S, Yaguchi A (2018). An investigation of the relationships between autistic traits and crossmodal correspondences in typically developing adults. Multisens. Res..

[46] Pellicano E, Burr D (2012). When the world becomes ‘too real’: A Bayesian explanation of autistic perception. Trends. Cogn. Sci..

[47] Van Boxtel JJ, Lu H (2013). A predictive coding perspective on autism spectrum disorders. Front. Psychol..

[48] Baron-Cohen S, Wheelwright S, Skinner R, Martin J, Clubley E (2001). The autism-spectrum quotient (AQ): Evidence from asperger syndrome/ high-functioning autism, males and females, scientists and mathematicians. J. Autism. Dev. Disord..

[49] Wakabayashi A, Baron-Cohen S, Wheelwright S, Tojo Y (2006). The autism-spectrum quotient (AQ) in Japan: A cross-cultural comparison. J. Autism. Dev. Disord..

[50] Bujang MA, Baharum N (2016). Sample size guideline for correlation analysis. World J. Soc. Sci. Res..

[51] Faul F, Erdfelder E, Lang AG, Buchner A (2007). G*power 3: A flexible statistical power analysis program for the social, behavioral, and biomedical sciences. Behav. Res. Methods..

[52] Wada M, Umesawa Y, Sano M, Tajima S, Kumagaya S, Miyazaki M (2022). Weakened Bayesian calibration for tactile temporal order judgment in individuals with higher autistic traits. J. Autism Dev. Dis..

[53] Kuroda, M., Inada, N. *Autism Diagnostic Observation Schedule*, Japanese Version (2nd Ed.). Kaneko Shobo, (in Japanese). (2015).

[54] Lord, C., Rutter, M., Dilavore, P.C., Risi, S., Gotham, K., Bishop, S.L. *ADOS: Autism Diagnostic Observation Schedule* (2nd Ed.). (Western Psychological Services, 2012).

[55] Fujita, K., Maekawa, H., Dairoku, K., Yamanaka, K. *A Japanese version of the WAIS-III*. (Nihon Bunka Kagakusha, 2006).

[56] Wechsler, D. *Wechsler Adult Intelligence Scale-III*. (The Psychological Corporation, 1997).

[57] Chen N, Tanaka K, Namatame M, Watanabe K (2016). Color–shape associations in deaf and hearing people. Front. Psychol..

[58] Wakabayashi A, Tojo Y, Baron-Cohen S, Wheelwright S (2004). The Autism-Spectrum Quotient (AQ) Japanese version: Evidence from high-functioning clinical group and normal adults. Jpn. J. Psychol..

[59] Morey, R.D., Rouder, J.N. BayesFactor: Computation of Bayes Factors for Common Designs. R package version 0.9.12–4.2. 2018.

[60] Ruzich E, Allison C, Smith P, Watson P, Auyeung B, Ring H, Baron-Cohen S (2015). Measuring autistic traits in the general population: A systematic review of the autism-spectrum quotient (AQ) in a nonclinical population sample of 6,900 typical adult males and females. Mol. Autism..

[61] R Core Team. R: a language and environment for statistical computing. Vienna, Austria: R Foundation for Statistical Computing (2020).

[62] Arend I, Naparstek S, Henik A (2013). Numerical-spatial representation affects spatial coding: Binding errors across the numerical distance effect. Psychon. Bull. Rev..

[63] Becker DV, Neel R, Anderson US (2010). Illusory conjunctions of angry facial expressions follow intergroup biases. Psychol. Sci..

[64] Goldfarb L, Treisman A (2010). Are some features easier to bind than others?: The congruency effect. Psychol. Sci..

[65] Hommel B, Colzato LS (2009). When an object is more than a binding of its features: Evidence for two mechanisms of visual feature integration. Vis. Cogn..

[66] Olivers CN (2012). Long-term visual associations affect attentional guidance. Acta Psychol..

[67] Rappaport, S.J. *Learning to overcome the binding problem* (Doctoral dissertation, University of Birmingham, 2013).

[68] Rappaport SJ, Humphreys GW, Riddoch MJ (2013). The attraction of yellow corn: Reduced attentional constraints on coding learned conjunctive relations. J. Exp. Psychol. Hum. Percept. Perform..

[69] Rappaport SJ, Riddoch MJ, Chechlacz M, Humphreys GW (2016). Unconscious familiarity-based color-form binding: Evidence from visual extinction. J. Cogn. Neurosci..

[70] Wildegger T, Riddoch J, Humphreys GW (2015). Stored color–form knowledge modulates perceptual sensitivity in search. Attent. Percept. Psychophys..

[71] Chen, N., & Watanabe, K. Color–shape associations in kids and parent-kid pairs. in *Proceedings of the Japanese Society for Cognitive Psychology: The 18th Conference of the Japanese Society for Cognitive Psychology*. p. 71 (2021).

[72] Chen N, Jiang X, Watanabe K. Color-shape association in Chinese people. In *11th International Conference on Knowledge and Smart Technology (KST)*. 209–212. (IEEE, 2019).

[73] Spector F, Maurer D (2008). The colour of Os: Naturally biased associations between shape and colour. Perception.

[74] Spector F, Maurer D (2009). Synesthesia: A new approach to understanding the development of perception. Dev. Psychol..

[75] Spector F, Maurer D (2011). The colors of the alphabet: Naturally-biased associations between shape and color. J. Exp. Psychol. Hum. Percept. Perform..

[76] Wagner K, Dobkins KR (2011). Synaesthetic associations decrease during infancy. Psychol. Sci..

[77] Ward J (2013). Synesthesia. Annu. Rev. Psychol..

[78] Maurer, D. Neonatal synesthesia: Implications for the processing of speech and faces. In: B. de Boysson-Bardies, S de Schonen, P Jusczyk, P McNeilage, J Morton (Eds.), *Developmental neurocognition: Speech and face processing in the first year of life*. 109–124. (Kluwer Academic, 1993)

[79] Stevenson RA, Siemann JK, Schneider BC, Eberly HE, Woynaroski TG, Camarata SM, Wallace MT (2014). Multisensory temporal integration in autism spectrum disorders. J. Neurosci..

[80] Mottron L, Dawson M, Soulieres I, Hubert B, Burack J (2006). Enhanced perceptual functioning in autism: An update, and eight principles of autistic perception. J. Autism. Dev. Disord..

[81] Stevenson RA, Siemann JK, Woynaroski TG, Schneider BC, Eberly HE, Camarata SM, Wallace MT (2014). Evidence for diminished multisensory integration in autism spectrum disorders. J. Autism. Dev. Disord..

[82] Belmonte MK, Yurgelun-Todd DA (2003). Functional anatomy of impaired selective attention and compensatory processing in autism. Cogn. Brain Res..

[83] Critchley HD, Daly EM, Bullmore ET, Williams SC, Van Amelsvoort T, Robertson DM, Murphy DG (2000). The functional neuroanatomy of social behaviour: Changes in cerebral blood flow when people with autistic disorder process facial expressions. Brain.

[84] Woods AT, Marmolejo-Ramos F, Velasco C, Spence C (2016). Using single colors and color pairs to communicate basic tastes II: Foreground–background color combinations. i-Perception.

[85] Ashby FG, Prinzmetal W, Ivry R, Maddox WT (1996). A formal theory of feature binding in object perception. Psychol. Rev..

