# Supplementary material for: People with higher autistic traits show stronger binding for color–shape associations
Source: Sci Rep. 2023 Jun 13;13:9611. doi: 10.1038/s41598-023-36666-4 (PMC10264394; doi:10.1038/s41598-023-36666-4)
Supplement: Supplementary file 1 — Supplementary Information. [file 41598_2023_36666_MOESM1_ESM.pdf]

Supplementary materials

Table S1. Profiles of participants with ASD

ID	NO.	Age	Sex	LQ	AQ	IQ			ADOS-2			Diagnosis
						Full	Verbal	Non-verbal	Communication	Social interaction	Total	
01	328	29	M	100	38	138	144	125	5	8	13	ASD
02	042	18	M	100	28	87	82	81	2	5	7	ASD/ADHD
03	055	25	M	-100	35	110	110	108	2	7	9	PDD
04	041	28	M	100	42	108	116	95	4	7	11	ASD
05	045	18	M	53	36	103	110	94	2	8	10	ASD/ADHD
06	061	22	F	89	44	119	109	129	4	7	11	ASD
07	049	21	M	100	38	120	130	102	3	6	9	PDD
08	060	32	M	100	25	91	99	83	3	9	12	ASD/ADHD
09	062	26	F	90	40	-	-	-	3	4	7	PDD
10	056	25	F	100	28	140	140	134	2	5	7	ASD

Note: LQ Laterality Quotient (Edinburgh Inventory); AQ Autism-spectrum Quotient; IQ Intelligence Quotient; ADOS-2 Autism Diagnostic Observation Schedule Component, Second Edition; ASD autism spectrum disorder; PDD pervasive developmental disorder; ADHD attention deficit and hyperactivity disorder.

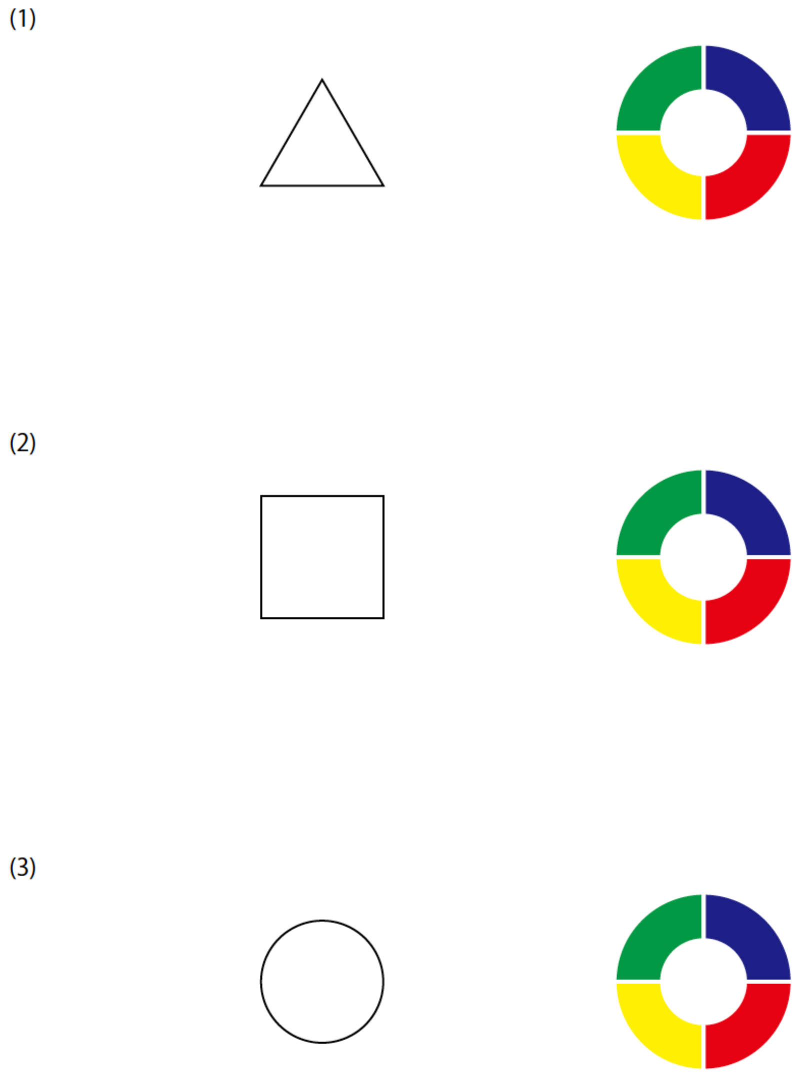

Figure S1. An example of explicit questionnaire survey on color-shape associations
